# Multimodality Imaging Supports Cardiac Lesion Diagnosis in Patient With Liver Carcinoma: A Case Report

**DOI:** 10.1002/cnr2.70088

**Published:** 2024-12-23

**Authors:** Hoa Thi Thuy Nguyen, Yen Thi Hai Nguyen, James N. Kirkpatrick, Viet Khoi Nguyen, Anh Van Nguyen, Hung Manh Pham, Walter Robert Taylor, Hoai Thi Thu Nguyen

**Affiliations:** ^1^ Vietnam National Heart Institute Bach Mai Hospital Hanoi Vietnam; ^2^ Department of Internal Medicine VNU‐University of Medicine and Pharmacy Hanoi Vietnam; ^3^ Cardiovascular Division, Department of Medicine, Department of Bioethics and Humanities University of Washington Medical Center Seattle Washington USA; ^4^ Radiology Center Bach Mai Hospital Hanoi Vietnam; ^5^ Department of Cardiology Hanoi Medical University Hanoi Vietnam; ^6^ Mahidol Oxford Tropical Medicine Research Unit Bangkok Thailand; ^7^ Centre for Tropical Medicine and Global Health University of Oxford Oxford UK

**Keywords:** cardiac magnetic resonance imaging, case report, liver adenocarcinoma, multimodality imaging, nonbacterial thrombotic endocarditis, three‐dimensional transesophageal echocardiography

## Abstract

**Introduction:**

Nonbacterial thrombotic endocarditis (NBTE) is a rare cardiac manifestation in patients with advanced malignancies of the lungs, pancreas, gynecological system, and gastrointestinal tract. It is often confirmed postmortem by histopathological evidence of sterile platelet‐fibrin deposits attached to the endocardium, most often on heart valves. To the best of our knowledge, our case is the first to report multiple heart lesions caused by the systemic effect of cholangiocarcinoma.

**Case Presentation:**

We report the case of a 53‐year‐old male who presented with a stroke; extensive imaging studies, including transthoracic echocardiography (TTE), 2D/3D transesophageal echocardiography (TEE), cardiac multi‐slice computed tomography, and cardiac magnetic resonance, found masses on the mitral valve, the aortic valve, and in the right ventricle, with the largest diameter 43 × 11 mm, which led to a diagnosis of NBTE secondary to presumed cholangiocarcinoma. Combining different echocardiography techniques, including TTE and TEE in specific clinical contexts, and training echocardiographers to improve TEE interpretation skills could be the most cost‐effective option for early diagnosis, particularly in limited‐resource settings, where advanced imaging modalities are not widely applicable.

**Conclusions:**

NBTE can manifest in patients with advanced cancer. A high index of clinical suspicion is of central importance for the diagnosis of NBTE, especially through an identification of the underlying predisposing conditions. A multi‐disciplinary approach is crucial for NBTE optimal diagnosis and treatment. As in our patient, multimodality imaging plays a complementary role in clearly defining the nature of cardiac lesions.

**Abbreviations**
^18^FDG‐PET/CT: 18‐fluorodeoxyglucose positron emission tomography/CT scan
3D: three‐dimensional
CMR: cardiac magnetic resonance imaging
LMICs: low‐ and middle‐income countries
MSCT: multi‐slice computed tomography
NBTE: nonbacterial thrombotic endocarditis
SPECT/CT: ^99^Technetium‐labeled leukocyte single photon emission computed tomography/CT scan
TEE: transesophageal echocardiography
TTE: transthoracic echocardiography

## 1 Introduction

Nonbacterial thrombotic endocarditis (NBTE), also known as sterile or marantic vegetation, often remains underdiagnosed in life due to a low index of suspicion. Moreover, confirmation of the diagnosis relies on histopathological evidence of a vegetation, namely, a sterile platelet‐fibrin deposit attached to the endocardium. Therefore, most diagnoses are established at postmortem examination [1]. Although some isolated NBTE cases have been found [2], NBTE is often associated with several diverse diseases such as systemic lupus erythematosus, antiphospholipid syndrome, rheumatic heart disease, HIV infection, and internal malignancies; the latter account for more than 50% of all NBTE cases [3].

NBTE is more common in solid organ tumors, notably, carcinomas of the lungs, pancreas, gynecological system, and gastrointestinal tract [4, 5]. It is more frequently reported in advanced malignancies, and thromboembolic complications, notably stroke, are often the first manifestation of cancer‐related NBTE [6, 7].

Malignancy‐related NBTE usually affects heart valves, primarily the mitral and aortic valves; simultaneous involvement of both valves is believed to be rare. To the best of our knowledge, there have been no reports of NBTE cases with involvement of the aortic and mitral valves and of the right heart [3, 4, 5, 8, 9].

The available data on NBTE are limited to small case series; thus, recommendations for managing these groups of patients depend on a limited body of evidence [1, 10]. However, the application of multiple imaging diagnostic tools is expected to support a definitive diagnosis, improve treatment, and prevent thromboembolic complications.

Herein, we present a case in which NBTE was diagnosed only after multimodality imaging examinations and a search for a primary tumor following exclusion of bacterial endocarditis. This case represents aortic and mitral valve vegetations and a concomitant right ventricle mass relating to carcinoma originating from the left lobe of the liver. Additionally, our case emphasizes the difficulty of confirming sterile vegetations. This highlights the necessity of applying multiple imaging modalities in this scenario to determine the comprehensive characteristics of cardiac masses in malignancy. In addition, in low‐ and middle‐income countries (LMICs), where advanced technologies are not widely applicable, a high index of suspicion combined with a stepwise approach and careful consideration of when to refer for less available and more expensive imaging studies are important.

## 2 Case Presentation

A 53‐year‐old male with a known history of untreated gallbladder stones first presented at another hospital with right hemiparesis secondary to an acute cerebral infarction noted on cerebral computed tomography. On further imaging work‐up, partial occlusion in distal branches of the right pulmonary artery was incidentally found without evidence of deep vein thrombosis. He was discharged with residual hemiparesis on 15 mg of daily rivaroxaban. On 8 January 2023, he presented to Bach Mai Hospital complaining of increasing hemiparesis and difficulty speaking; he did not have a fever, chest pain, or dyspnea. He had a pulse of 82 beats per minute, a blood pressure of 130/80 mmHg, mild jaundice, a grade 3 systolic murmur best heard over the apex of the heart, and hepatomegaly. He did not have retinal Roth spots, splinter or conjunctival hemorrhages, Osler's nodules, or Janeway's lesions. He had a motor dysphasia and a hemiparesis of 3/5 on the Medical Research Council scale for muscle strength.

**FIGURE 1:**
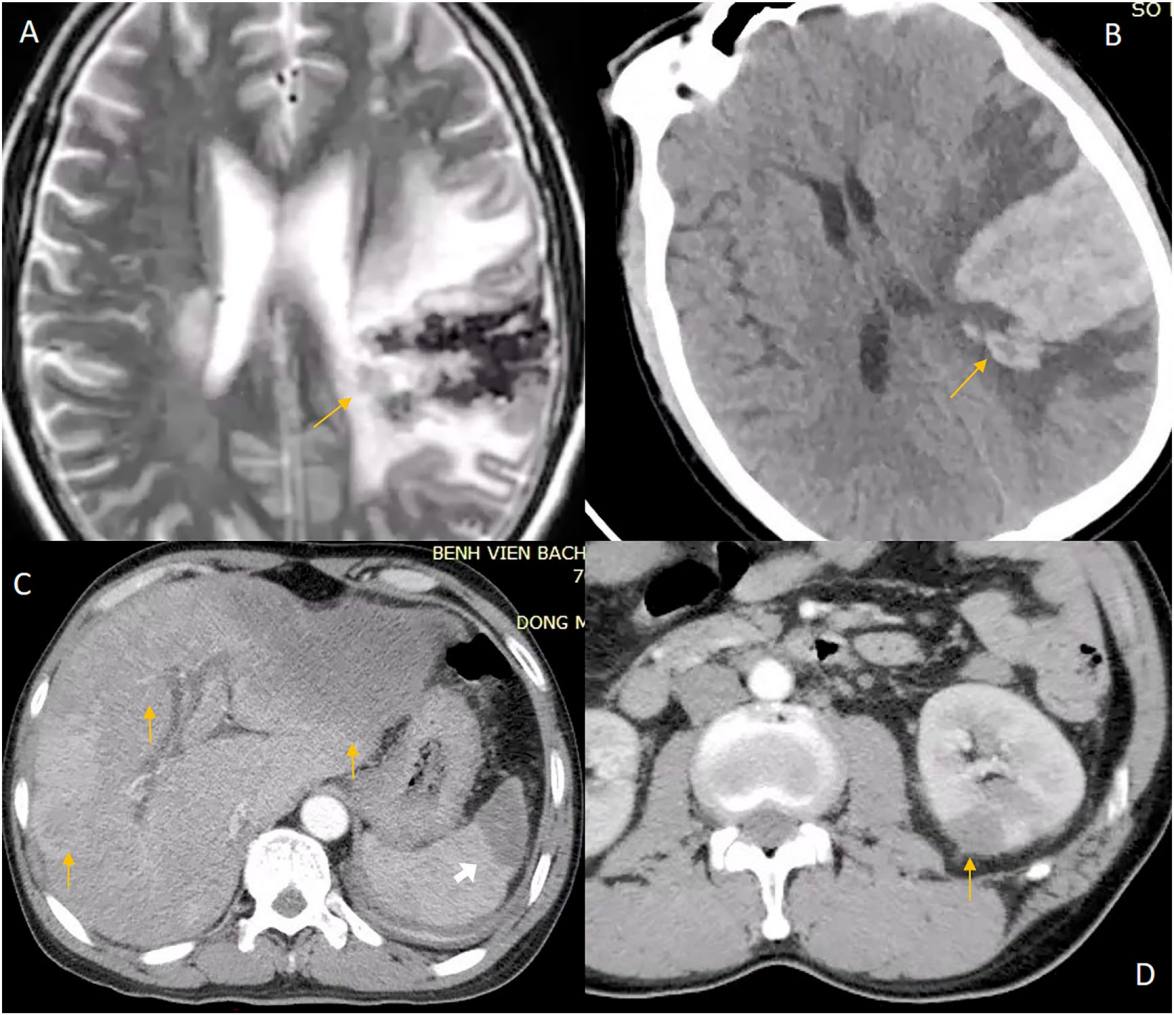
Extracardiac manifestations. (A) T2 weighted magnetic resonance imaging showing cerebral infarction with hemorrhagic transformation. (B) The same lesion on non‐contrast CT. Abdominal multi‐slice computed tomography (arterial phase) image showing: (C) Hypointense masses in left and right lobes of the liver (yellow arrows) and spleen 535 (white arrow). (D) Hypointense mass in the left kidney.

Magnetic resonance imaging (MRI) of the brain showed a left lateral periventricular infarction with hemorrhagic transformation (Figure 1A). The hematoma was 30 × 40 mm in size and was located in the left temporal lobe without gadolinium uptake in the lesion. This new hemorrhage correlated with his symptoms.

**FIGURE 2:**
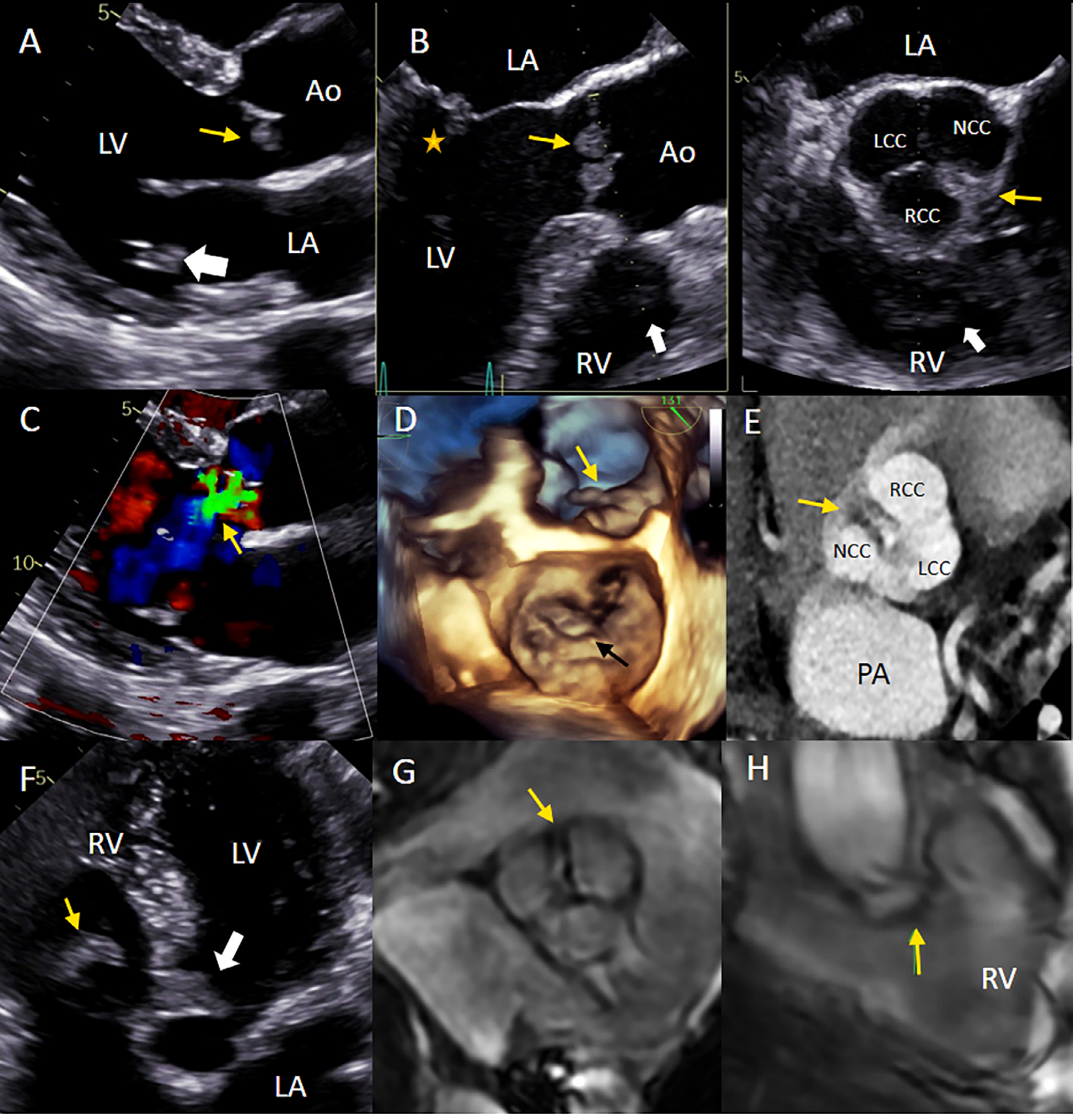
Cardiac masses on different imaging modalities. (A) TTE parasternal long axis view showing aortic valve masses on both cusps (yellow arrow) and a mitral valve mass on the posterior leaflet (white arrow). (B) TEE X‐plane image from the mid‐esophageal position, left: AV long axis view showing aortic valve masses (yellow arrow), and a mitral valve mass on the posterior leaflet (star), and right ventricular mass (white arrow), right: AV short axis view showing cardiac masses on all cusps of the aortic valve (yellow arrow) and right ventricular mass (white arrow). (C) Aortic regurgitation on 2D TTE. (D) 3D TEE enface view showing the mitral valve mass (black arrow) and aortic mass (yellow arrow). (E) Aortic mass (yellow arrow) on 3 cusps of the aortic valve on cardiac MSCT. (F) TTE apical 5 chamber view showing right ventricular mass (yellow arrow) and aortic valve mass (white arrow). (G) CMR image showing aortic mass on cine imaging (arrow). (H) CMR image showing the right ventricular mass (arrow). Ao: Aorta, LA: Left atrium, LCC: Left coronary cusp, LV: Left ventricle, PA: Pulmonary artery, RV: right ventricle, NCC: Non‐coronary cusp, RCC: Right coronary cusp.

Routine transthoracic echocardiogram (TTE) revealed an aortic valve vegetation‐like mass, measuring 10.7 × 13.8 mm, with a homogenous, slightly hypoechoic texture; this mass was attached to the edge of the right coronary cusp and non‐coronary cusp on the left ventricle outflow tract side, where it displayed restrictive movements during the cardiac cycle and was associated with mild aortic regurgitation. There was a second vegetation‐like mass attached to the atrial side of the posterior leaflet of the mitral valve, measuring 11 × 4.4 mm. There was a mild mitral regurgitation. A right ventricular mobile lesion, measuring 43 × 11 mm with similar echogenicity, was also seen (Figure 2A,C).

A 3D transesophageal echocardiogram (TEE) was performed to confirm these findings, and we detected another lesion attached to the left coronary cusp of the aortic valve. The masses were seen on both the upstream side and the downstream side of the aortic valve. The right ventricular mass, which was not well observed on TTE, was clearly visualized on TEE. The mass was hypoechoic, shaped like a band, and attached to the moderator band of the right ventricle. 3D real‐time TEE imaging from the mid‐esophageal position allowed a biplane evaluation of the aortic valve and views of all masses in the short and long axis (Figure 2B) with good characterization of the motion of the masses (Figure 2D).

To allow for greater anatomical discrimination, cardiac MSCT revealed hypointense lesions on all 3 aortic valve leaflets, measuring 7 × 13, 4 × 15, and 4 × 6 mm, which had a homogeneous texture, lacked contrast enhancement, and demonstrated little movement during the cardiac cycle (Figure 2E). The coronary arteries were normal. A cardiac magnetic resonance (CMR) imaging study was performed, but the patient's stroke limited his ability to hold his breath, so we resorted to a free‐breathing CMR. The right ventricular lesion appeared as a band‐shaped hypointense signal on cine images, without gadolinium enhancement, that arose from the right ventricular moderator band and extended to the tricuspid annulus. It was hypointense on T2‐weighted images and hyperintense on fat‐suppressed T2‐weighted images (Figure 2G,H).

Three sets of blood cultures collected within 24 h before antibiotic initiation were negative for bacterial and fungal growth, and the PCR was negative for the detection of Coxiella burnetti, Aggregatibacter sp., Cardiobacterium sp., *Staphylococcus aureus* , Enterococcus sp., *Streptococcus suis* , Streptococcus pneumonia, other Streptococcus sp., *E. coli* , Klebsiella pneumonia, *Pseudomonas aeruginosa,* and Aspergillus. The protein C, protein S, and anti–thrombin III results were within the normal limits; D‐dimer levels were > 7.65 mg/L FEU (< 0.5), as were the PT at 21.8 s, and APTT at 42.5 s. Anti‐double‐stranded DNA and ANA antibodies were negative.

**FIGURE 3:**
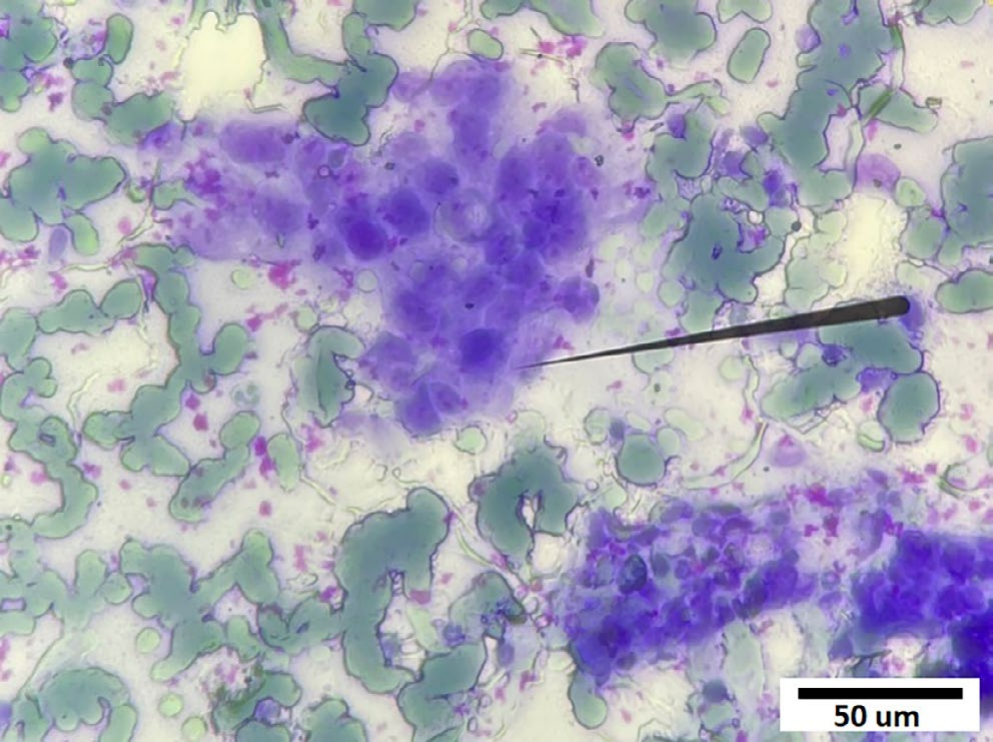
Fine needle aspiration: Cluster of abnormal cells with enlarged alkaline nuclei and high nucleocytoplasmic ratio (Hematoxylin—Eosin, ×400).

A liver ultrasound detected a lesion in the left lobe that on abdominal MSCT appeared as a hypointense mass, 12.1 × 5.2 cm, that almost completely occupied the left lobe; it had a coarse capsule and contained dilated bile ducts. Hepatic capsule retraction was noted, but capsule penetration was not observed. After contrast agent injection, the tumor was hypovascularized in the arterial phase. All of these characteristics are consistent with cholangiocarcinoma. There were several nodes in the right lobe of the liver with similar imaging features; the largest node was 1.3 cm in diameter (Figure 1C). In addition, a CT scan showed a stone in the common hepatic duct measuring 1.5 cm in diameter and groups of stones, measuring 1.5 × 0.5 cm, in the common bile duct but without bile duct dilation. Hypointense masses were also detected in the spleen and kidney. These masses remained hypointense after contrast agent administration (Figure 1C,D). There was mild free intraperitoneal fluid. Additional laboratory tests reported an alpha fetoprotein (AFP) concentration of 2.8 ng/mL (< 7.0), a remarkably high cancer antigen concentration: CA 19–9: 1000 U/mL (< 27), CA 12–5: 1679 U/mL (< 35), and a carcinoembryonic antigen (CEA) concentration of 1000.0 ng/mL (< 4.3). Owing to his elevated PT and APTT, a liver biopsy could not be performed, but he underwent a fine needle aspiration of the liver mass; histological examination confirmed a carcinoma but lacked specificity determining the tissue origin (Figure 3).

**FIGURE 4:**
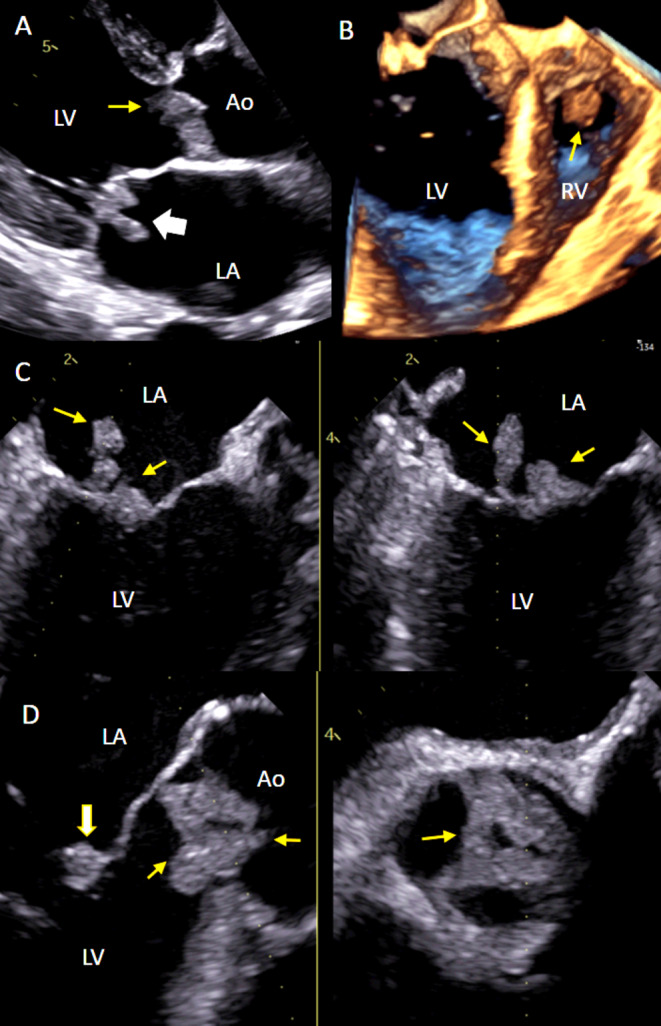
Follow‐up TTE and TEE. (A) TEE parasternal long axis view showing aortic valve masses on both cusps (yellow arrow) and mitral valve mass on both leaflets (white arrow). (B) 3D TEE showing right ventricular mass (yellow arrow). (C) TEE X‐plane image from the mid‐esophageal position, left: Long axis view, right: Mitral commissural view showing mitral valve masses on both leaflets (yellow arrows). (D) TEE X‐plane image from the mid‐esophageal position, left: AV long axis view showing cardiac masses on all cusps of aortic valve (yellow arrow) and mitral valve mass on the anterior leaflet (white arrow), right: AV short axis view showing aortic valve masses (yellow arrow). Ao: Aorta, LA: Left atria, LV: Left ventricle, RV right ventricle.

**FIGURE 5:**
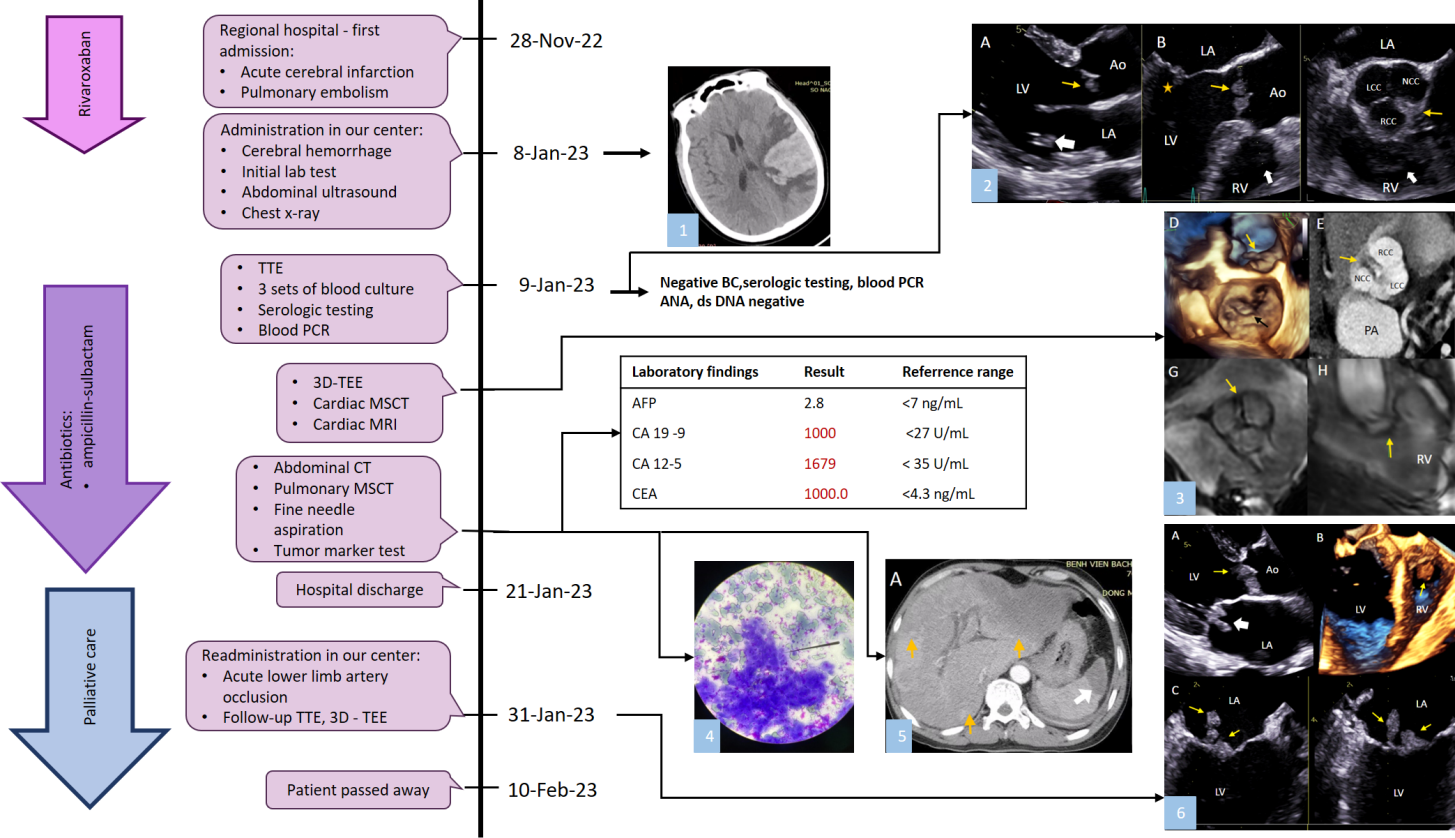
Timeline of patient clinical course. (1) Cerebral infarction with hemorrhagic transformation observed on non‐contrast CT. (2) A—TTE parasternal long‐axis view showed masses on both aortic valve cusps (yellow arrow) and a mitral valve mass on the posterior leaflet (white arrow). B—TEE X‐plane imaging from the mid‐esophageal position; left: AV long‐axis view showed aortic valve masses (yellow arrow), a mitral valve mass on the posterior leaflet (star), and a right ventricular mass (white arrow); right: AV short‐axis view showed cardiac masses on all cusps of the aortic valve (yellow arrow) and the right ventricular mass (white arrow). (3) D—3D TEE en face view showed the mitral valve mass (black arrow) and aortic valve masses (yellow arrow). E—Cardiac MSCT revealed aortic masses (yellow arrow) on all three cusps of the aortic valve. F—TTE apical five‐chamber view showed the right ventricular mass (yellow arrow) and the aortic valve masses (white arrow). G—CMR cine imaging showed the aortic masses (arrow). H—CMR showed the right ventricular mass (arrow). (4) Fine needle aspiration. (5) A—Hypointense masses identified in the left and right liver lobes (yellow arrows) and in the spleen (white arrow) during the arterial phase of abdominal MSCT. (6) A—TTE parasternal long‐axis view showed aortic valve masses on both cusps (yellow arrow) and the mitral valve mass on both leaflets (white arrow). B—3D TOE showed the right ventricular mass (yellow arrow). C—TEE X‐plane imaging from the mid‐esophageal position; left: long‐axis view showed mitral valve masses on both leaflets (yellow arrows); right: mitral commissural view also showed mitral valve masses on both leaflets (yellow arrows).

A MSCT of the pulmonary artery revealed partial or complete occlusion of several segmental branches and consolidation of the laterobasal segment of the right lower lobe. Ultrasonography of abdominal arteries was unremarkable.

After multidisciplinary team consultation, the patient was diagnosed with NBTE associated with advanced cholangiocarcinoma with multiple embolic complications. Then he was discharged to receive home‐based palliative care. No anticoagulation agent was prescribed because of his recent cerebral hemorrhage that was likely exacerbated by his rivaroxaban. Ten days later, he suffered an acute lower limb artery occlusion and was readmitted. An echocardiogram showed that all the valvular and the ventricular masses had increased in size and that the degree of mitral and aortic regurgitation had progressed (Figure 4). TEE with 3D real‐time narrow‐angle imaging confirmed the right ventricular mass and its anatomical characteristics. The patient died 5 days later.

## 3 Discussion

The pathological mechanism of NBTE in patients with malignancies has not been fully elucidated; NBTE is believed to arise from a hypercoagulable state and endothelial damage secondary to cancer [11]. Malignant cells have specific impacts on the coagulation cascade through the ability to create active coagulant precursors, activate the fibrinolytic system, secrete inflammatory cytokines, and directly interact with platelets, monocytes, or endothelial cells [12]. Mucin‐secreting adenocarcinomas in the lungs, pancreas, gynecological system, and gastrointestinal tract are most frequently associated with NBTE [4]. Other well‐known causes include SLE, antiphospholipid syndrome, and tuberculosis. The prevalence of NBTE reported at postmortem examination varies between 0.9% and 1.6% [3] but higher rates have been reported in echocardiography studies. The classic diagnosis of NBTE relies on the detection of a heart murmur and multiple systemic emboli in a patient with an underlying NBTE‐inducing disease [13]. Our patient was admitted with the typical NBTE presentation of thromboembolic events [14]. Cardiac vegetation‐like masses were incidentally found on routine work‐up (Figure 5).

An increasing number of NBTE cases have been reported in recent years, reinforcing the understanding of this disease in the medical literature [4]. Accordingly, NBTE is most frequently found on the aortic valve, followed by the mitral valve. Cases with multivalve involvement are considered to be unusual [11], and NBTE affecting both sides of the heart is extremely rare [15, 16]. We believe this is the first case of cholangiocarcinoma‐related NBTE with multiple intracardiac vegetation‐like masses affecting the aortic valve, mitral valve, and right ventricle. Furthermore, our case emphasized the multiple imaging modalities approach, in which we attempted to gather potentially imaging features to aid in NBTE diagnosis.

Despite the availability of multiple imaging modalities to support cardiac investigations, antemortem diagnosis of NBTE remains challenging. We suggest that TTE and TEE are the mainstay imaging modalities, especially in patients with severe illness and numerous lesions in different cardiac chambers, as in our case. TTE is a very useful and widely available test and should be the first imaging technique to be performed [16]. However, its limitations include difficulty in obtaining optimal views in some patients and a lower sensitivity for endocarditis compared to TEE [17]. Our patient's TEE resulted in good image quality and detected other lesions, including the single, small vegetation on the left coronary cusp of the aortic valve, missed on TTE. TEE also detected vegetations on both the upstream side and downstream side of the aortic valve, indicating a very high risk of embolization. Moreover, TEE provides excellent spatial resolution and good views of intra‐cardiac structures.

TTE and TEE are commonly recommended as first‐line imaging modalities for diagnosing infective endocarditis [10], and both should be considered in suspected NBTE [16]. Furthermore, 3D TEE, which is also valuable in infective endocarditis [10], should also be applied to diagnose NBTE because it provides more detailed information than 2D imaging, albeit at a lower effective resolution. In our case, 3D TEE provided comprehensive and detailed imaging of the cardiac structures and masses. However, TTE and 3D TEE are performer dependent and lack sufficient specificity in tissue characteristic assessment [18].

On echocardiography, NBTE lesions are small, typically < 1 cm in diameter, broad‐based and irregular in shape [19]; they are hypoechogenic with a homogenous consistency, and NBTE vegetation does not cause valvular perforation or degeneration so that valve regurgitation is usually mild, at most moderate. The echocardiographic features that favor benign from malignant cardiac masses include greater mobility, adherence to the interatrial septum, and the presence of a pedicle. On the other hand, infiltration, significant pericardial effusion, right‐sided localization, sessile implantation, polylobate shape, and heterogeneous consistency are associated with malignancy of the mass [20].

One important differential diagnosis of NBTE is cardiac thrombus. Histologically, NBTE is a special type of thrombus, consisting primarily of degenerated platelets and fibrin [19, 21]. Therefore, NBTE should have similar imaging characteristics of thrombus, including heterogeneous signal intensity on CMR LGE. An acute thrombus presents as an isointense signal on CMR T1‐weighted images and a hypointense signal on T2‐weighted images, which becomes hyperintense on T1‐weighted images and hypointense on T2‐weighted images in the transition to a subacute thrombus. In contrast, an organized chronic thrombus will appear with a low signal intensity on both T1 and T2 weighted images and possibly include a white ring on LGE [22]. Differentiating NBTE from infective vegetation is sometimes a challenge for the clinician. CMR with its LGE sequences is able to help characterize thrombus [23, 24, 25]. CMR provides excellent tissue characterization and can detect delayed enhancement, representing endothelial inflammation, supporting a diagnosis of infective endocarditis over NBTE or thrombus [26]. However, CMR has low spatial resolution, so it may miss small mobile masses [18]. A typical CMR protocol requires patients to be able to hold their breaths; this was too challenging in our patient, and we were only able to obtain T2‐weighted and fat‐suppressed T2 images of his heart. On T2‐weighted images, the right ventricular mass appeared homogenous and hypointense compared to normal myocardium with no sign of infiltration, but we were unable to glean useful information pertaining to vegetation size and motion. However, there was no other imaging evidence to support a diagnosis of metastasis or primary cardiac tumor (Table 1) [27, 28].

**TABLE 1:** CMR features differentiate between malignant and benign cardiac masses [27, 28].

CMR features	Thrombus	Tumor	Tumor	
			Benign	Malignant
Pre‐contrast visualization	+++	++++	++++	++++
Multiple	+/−	+/−	+/−	+/−
Homogeneous	++++	+	+	++
Mobility	+/−	++	++	++
T1w‐TSE hyper‐intensity	+/−	+/−	+	+/−
T2w‐TSE hyper‐intensity	+	+++	+++	+++
FPP (+)	−	++	+	+++
LGE (+)	+/−	++	+	+++
Typical TI scout pattern	+++	−	+/−	−
Median diameter	Thrombus < Tumor		Benign tumor < Malignant tumor	
“++++”: account for > 95% of cases, “+++”: 75%–95%, “++”: 50%–75%, “+”: 25%–50%, “+/−”: 5%–25%, “–”: < 5%; FPP: first‐pass perfusion, T1w‐TSE: T1 weighted turbo‐spin echo, T2w‐TSE: T2 weighted turbo‐spin echo, TI: inversion time				
	Benign mass		Malignant mass	
Polylobate	−		+	
Non‐left localization	−		+	
Sessile	−		+	
Infiltration	−		++	

Pericardial effusion	−	+
“+”: more likely; “−”: less likely		

On the other hand, cardiac CT provides optimal anatomical evaluation, so valvular lesions can be clearly observed, and CT is an alternative for patients with CMR contraindications, small valvular masses, or who cannot perform breath holding. It is important to plan the injection protocol because different protocols are needed for left‐ and right‐sided lesions [29]. Our patient had bilateral lesions but underwent the regular CT protocol, so only his left‐sided lesions were well imaged because of right‐sided contrast flow artifacts. Nuclear imaging, including 18‐fluorodeoxyglucose positron emission tomography/CT scan (^18^FDG‐PET/CT) and ^99^Technetium‐labeled leukocyte single photon emission computed tomography/CT scan (SPECT/CT), has been added to the infective endocarditis investigation protocol to improve diagnostic sensitivity. Inflammation in the endothelium can be detected before anatomical alterations can be recognized [25, 30]. If this procedure could have been performed on our patient, it would eliminate the occurrence of any metabolic activities relating to the patient's cardiac masses, therefore establishing the diagnosis of NBTE [30]. Limitations are low spatial resolution (limited to ≥ 5 mm) in comparison with TEE or CT scans and lower specificity [25].

Access to imaging is crucial for the diagnosis and treatment of NBTE patients. Nevertheless, there have been shortages of imaging equipment and workforce in LMICs. Reasons explaining the insufficient use of advanced technologies in LMICs despite their widespread use in high‐income countries include the lack of investment plans and prioritization, the cost of purchasing equipment, and long‐term maintenance of the equipment. Additionally, a properly trained workforce is needed to operate CT and CMR scanners. On the other hand, echocardiography, due to its low price, its being simple to be maintained, and its high practical yield, is the most used imaging modality in LMICs [31]. Although referral centers in LMICs may have access to 3D TEE, cardiac CT, and CMR, many places only have 2D echocardiography. Therefore, echocardiography features and the clinical context play an important role in referral decisions. However, referral often comes with high transportation costs. We need more training in resource‐limited settings about NBTE in order to make sure that an appropriate index of suspicion is maintained. Echocardiography must be performed as soon as there is a clinical suspicion. Non‐infective masses on echocardiography can be suspected in the presence of small and multiple lesions, changing from one echo exam to another. Multiple nonstandard views are needed because vegetation may be visualized in off‐axis views and be tiny, especially in the early phase of the disease. In addition, training medical staff to improve TEE interpretation skills is a cost‐effective approach to enhancing diagnostic accuracy. We also need more studies to correlate findings on more widely available imaging studies such as echocardiography with those on more expensive and limited studies such as PET/CT and SPECT/CT. For instance, the use of ultrasound‐enhancing agents has been shown to identify vascularity in tumors and might serve in a triage role in limited resource settings [32, 33, 34].

Our case reveals the fact that the diagnosis and treatment of NBTE are still influenced by local availability and expertise. This case emphasizes the importance of a multi‐disciplinary approach involving oncologists, cardiovascular imaging specialists, and pathologists in diagnosing and managing patients with NBTE. The diagnosis of NBTE was missed during the initial hospitalization, leading to inappropriate use of anticoagulation. As a result, NBTE continued to progress, and the risk of cerebral hemorrhage transformation increased. Although non‐vitamin K oral anticoagulants have shown potential in the treatment of cancer‐associated thrombosis [35], it had been reported to cause negative effects when used in NBTE patients [36, 37]; no evidence supports the use of direct oral anticoagulants in NBTE settings. Vitamin K antagonists are less efficacious than heparin for the prevention of thromboembolic events in cancer‐related NBTE. Heparin inhibits the effect on factor Xa and thrombin and has beneficial effects in cancer thrombosis, including increased release of tissue factor pathway inhibitor from the vascular endothelium at sites of thrombosis, binding and neutralizing cytokines and chemokines, hence preventing microthrombi formation [38, 39]. Therefore, the mainstay of managing patients with cancer‐related NBTE is underlying malignancy treatment and low molecular weight heparin [1, 36].

Multiple cardiac imaging modalities, especially TEE with 3D acquisition, provided comprehensive information on all of the patient's cardiac masses. We also recommend that physicians consult with a medical center that has multimodality imaging when investigation results are inconclusive. Follow‐up protocol should include both clinical and imaging evaluation, as it is of importance to search for recurrent embolism and ensure appropriate antithrombotic therapy as well as minimize the risk of bleeding and thrombocytopenia.

## 4 Conclusion

NBTE is often associated with malignancy and underdiagnosed. Embolic complications are not infrequent. Diagnosis and treatment are still influenced by local availability and expertise. A high index of clinical suspicion, especially in the context of underlying predisposing conditions, is of central importance to detect earlier incidence before the development of thromboembolic complications. A multi‐disciplinary approach is fundamental for its optimal evaluation and management. Multimodality imaging plays a complementary role in clearly defining the nature of cardiac lesions. In a limited resource setting, training medical staff to improve TEE skills is a cost‐effective approach to enhance diagnostic accuracy.

## 5 Patient's Perspective

I have been ordered to perform many imaging investigations, which have not been done at previous medical centers. Doctors showed me the results and explained them to me in detail. Finally, I feel thankful that they determined the cause of my problems.

## Author Contributions

**Hoa Thi Thuy Nguyen:** conceptualization, investigation, writing – original draft. **Yen Thi Hai Nguyen:** investigation, writing – original draft. **James N. Kirkpatrick:** conceptualization, methodology, writing – original draft, writing – review and editing. **Viet Khoi Nguyen:** investigation, writing – original draft. **Anh Van Nguyen:** investigation, writing – original draft. **Hung Manh Pham:** investigation, writing – original draft, conceptualization. **Walter Robert Taylor:** conceptualization, investigation, writing – original draft, writing – review and editing, methodology. **Hoai Thi Thu Nguyen:** conceptualization, investigation, methodology, writing – original draft, writing – review and editing, visualization, supervision, resources.

## Consent

Written informed consent was obtained from the patient for publication of this case report and any accompanying images. A copy of the written informed consent is available for review by the editor‐in‐chief of this journal.

## Conflicts of Interest

The authors declare no conflicts of interest.

## Data Availability

The authors confirm that the data supporting the findings of this study are available within the article [and/or] its Supporting Information.
